# Temperament and Character Profiles and Psychiatric Comorbidities in Patients With Coronary Artery or Valvular Heart Disease: Relationship With Cardiac Disease Severity

**DOI:** 10.14740/jocmr2440w

**Published:** 2016-01-26

**Authors:** Cigdem Hazal Bezgin, Tahir Bezgin, Sermin Kesebir

**Affiliations:** a Department of Psychiatry, Erenkoy Psychiatry Training and Research Hospital, Goztepe, Istanbul, Turkey; b Department of Cardiology, Kartal Kosuyolu Heart Research Hospital, Kartal, Istanbul, Turkey

**Keywords:** Coronary artery disease, Valvular, Temperament and character, Psychiatric symptoms

## Abstract

**Background:**

We aimed to investigate whether the psychopathological symptoms and temperament-character dimensions observed in patients operated due to coronary artery disease (CAD) or valvular heart disease (VHD) differ among the patients and from healthy individuals.

**Methods:**

Study population was composed of subjects with CAD, VHD and healthy controls (n = 50 in each group). Socio-demographic questionnaire, temperament and character inventory (TCI) and symptom check list-90-R (SCL-90-R) were applied to all groups. Groups were compared about temperament-character dimensions and scores of subscales of SCL-90-R.

**Results:**

Harm avoidance was found to be higher in VHD group than those with CAD and, lower in healthy controls than both patient groups (P = 0.004). Reward dependence was similar among both patient groups and, was higher than healthy group (P = 0.015). Depression, anxiety, somatization, obsession and interpersonal sensitivity were found to be similar in both patient groups but they were higher than those in controls (P < 0.001, P < 0.001, P < 0.001, P = 0.002 and P = 0.003, respectively). Phobia was seen equally in CAD group and healthy controls and, was found to be lower in these than in VHD (P = 0.009). Anger score was in descending order in patients with VHD, CAD and healthy controls group (P = 0.010 and 0.001). Paranoia was in descending order in patients with VHD, CAD and controls (P = 0.015 and 0.001). A weak and inverse correlation was found between ejection fraction (EF) and the persistence dimension of temperament scaled by TCI in patients with VHD (r = -0.276, P = 0.052). An inverse correlation was observed between EF and the reward dependence dimension in CAD group (r = -0.195, P = 0.044). In patients with VHD, EF demonstrated an inversely weak (r = -0.289, P = 0.042), moderate (r = -0.360, P = 0.010) and strong (r = -0.649, P < 0.001) correlation with inter-personal sensitivity, phobia and paranoia, respectively. There was an inverse and weak correlation between EF and depression and anger in VHD group (r = -0.302, P = 0.033 and r = -0.240, P = 0.054).

**Conclusion:**

VHD and CAD exhibit different psychopathological symptoms and temperament traits. There is a correlation between the aforementioned psychopathological symptoms and temperament traits, and EF.

## Introduction

Up-to-date data evidence has been obtained regarding the correlation of coronary artery disease (CAD) with psychological factors in the studies by Friedman [1] and Rosenman et al [2] and these investigators have described the coronary-associated behavioral pattern. This behavioral pattern included competitiveness, restiveness, aggression, hostility, feeling of lack of time, pathological control efforts, ambition, anger, clenching, excessively rapid body movements, strained face and body muscles, explosive speech and setting excessively high performance standards [3].

The psychosocial and behavioral factors including mood (anxiety, depression and stress), personality traits (type A and type D and hostility) and social support are not only related to the development but also to the progression of cardiovascular disorder. Mood and personality traits are the first two of the five main titles listing the psychological and behavioral risk factors that affect the development of CAD [4]. Depression and anxiety have been associated with both the development and progression of CAD, irrespective of biological risk factors such as obesity, hypertension, diabetes, dyslipidemia and insulin resistance [5-10]. The personality traits associated with CAD have been described under the type A and D personality profile.

In contrast to the rich literature on CAD, this subject is a rarely investigated field except the correlation between the valvular heart diseases (VHDs) and depression and anxiety, and there are no relevant systematic studies. An unfavorable effect of depression has been reported with long-term results in patients who underwent valvular surgery [6]. Major depressive disorder and post-traumatic stress disorder were found to increase in-hospital mortality risk following valvular surgery [11]. The observational studies evaluating patients who underwent valvular surgery with cases of coronary bypass reported an association between anxiety and increased mortality and cardiovascular morbidity.

In this study, patients with CAD or operated VHD and no previous diagnosis of psychiatric disorder in whom the ejection fraction (EF) was calculated by transthoracic echocardiography as an indicator of disease severity were compared to healthy controls with no cardiovascular, valvular or diagnosed psychiatric disorders. The symptoms check list-90-R (SCL-90-R), and the temperament and character inventory (TCI, Turkish) were applied to all participants.

Our first hypothesis is that the psychopathological symptoms and temperament dimensions observed in patients with CAD and VHD differ among the patients and from those observed in healthy individuals. Our second hypothesis is that the severity of CAD and VHD correlates with the severity of psychological symptoms and temperament traits.

## Methods

This case-control multi-center study was composed of patients with CAD, operated VHD and age- and sex-matched healthy controls. Patients gave written informed consent in accordance with the Declaration of Helsinki. The Institutional Review Board of Erenkoy Psychiatry Training and Research Hospital approved the study.

### Patients and controls

The study sample consisted of 50 patients with a history of CAD or 50 patients with operated VHD who presented at the outpatient Clinic of Kartal Kosuyolu Heart Research Hospital. The healthy control arm consisted of healthy hospital employees with no diagnosed medical disorder or cardiac pathology.

Exclusion criteria were: 1) being younger than 18 and older than 65 years of age; 2) presence of mental retardation, dementia, delirium or other amnestic disorders at a level detectable by medical assessment; 3) previously diagnosed, currently on-treatment or untreated psychiatric disorder; and 4) chronic disorders except diabetes mellitus (DM) and hypertension (HT), and other cardiac diseases except coronary and/or VHD (arrhythmia, idiopathic dilated cardiomyopathy, hypertrophic cardiomyopathy, etc.).

Patients diagnosed with CAD or VHD who presented at the Outpatient Clinic of Kartal Kosuyolu Research Hospital, and the healthy control group were evaluated via echocardiography. Echocardiographic assessments were conducted by a single physician (T.B.), using a 2.5 - 3.5 MHz phased-array transducer probe (GE Vingmed, Vivid 3). Echocardiograms were obtained in left lateral decubitus position and from standard echocardiographic windows (parasternal long and short axis, apical two- and four-chamber). Two-dimensional and M-mode measurements were performed as per the requirements of the American Echocardiography Society guidelines [12]. Left ventricular end-diastolic volume (EDV), end-systolic volume (ESV) and EF were measured from the apical two- and four-chamber images using the modified Simpson’s method.

The socio-demographic data collection form, TCI and SCL-90-R were applied to all participants.

### Statistical analysis

The statistical analyses were performed using the SPSS (Statistical Package for the Social Sciences) 16 software. Kolmogorov-Smirnov test was used to check whether the variables were distributed normally. Upon an observation of a normal distribution, χ^2^ test and ANOVA test (analysis of variance) were used respectively to compare categorical variables and numeric variables. To determine the group of the variables detected to be significantly different based on the intergroup comparison, *post hoc* χ^2^ test and *post hoc* Benferroni test were used for the categorical and numeric variables, respectively. The correlation analysis was performed with Pearson’s correlation test. P < 0.05 was considered significant, and all tests were two-tailed.

## Results

The socio-demographic and medical history characteristics are summarized in Table 1 for CAD patients, VHD patients, and healthy individuals.

**Table 1:** Description of the Sample

	VHD (N = 50)	CAD (N = 50)	Healthy controls (N = 50)	Analysis	
				χ^2^/F	P
Age (mean ± SD)	50.9 ± 5.9	50.6 ± 6	51 ± 6	0.043	0.958
Gender (F/M)	38/12	38/12	38/12	0	NS
Years of education (mean ± SD)	8.4 ± 3.7	10.3 ± 4.6	10.4 ± 5.1	2.852	0.007
Marital status (married)	50	49	50	0.029	0 .996
Employed (%)	52	52	52	0	NS
History of psychiatric disorder (%)	-	-	-		
Familial history of psychiatric disorder (%)	18	4	6	6.775	0.034
DM (%)	4	12	4	429	0.180
HT (%)	12	14	2	4.884	0.048
Familial history of cardiac disorder (%)	28	50	30	6.424	0.040
Smoking (%)	32	44	38	7.116	0.310
Alcohol (%)	-	4	-		
Ejection fraction (%) (mean ± SD)	59.1 ± 9.8	55.8 ± 9.9	65 ± 1	16.374	< 0.001

Bonferroni correction was applied for the *post hoc* analysis. For EF, VHD = CAD < HC.

The mean age was 50.9 ± 5.9, 50.6 ± 6 and 51 ± 6 years in VHD, CAD and control groups, respectively. The mean age showed similarity between the two patient groups and the healthy control group (P = NS). Comparison of the gender rates between the groups (P = NS) revealed no difference: VHD (F: 76%; M: 24%), CAD (F: 76%; M: 24%), healthy control group (F: 76%; M: 24%). The mean number of education years was observed to be lower in the VHD group relative to CAD and healthy control groups. The comparison of employment status between the groups revealed no significant difference (P = NS). The groups exhibited no difference with respect to smoking and alcohol use (P = NS).

While no history of psychiatric disorder was detected in any of the three groups, the incidence of familial history of psychiatric disorder was higher in the VHD group. While diabetes was more common among the patients with CAD (12%), HT was more common in both patient groups compared to the healthy controls. The incidence of familial history of cardiac disease was similar between the VHD patients and the healthy controls, and higher in the CAD group.

There was a significant difference between the groups with respect to EF (P < 0.001). The Bonferroni correction applied to determine from which group this difference resulted, revealed a significant difference between the VHD group and healthy controls (P < 0.001), and between the CAD group and healthy controls (P < 0.001), while there was no significant difference between VHD and CAD groups (P = NS).

### Comparison of temperament-character and SCL-90-R dimensions between the groups

Temperament-character dimensions were compared between the patients with VHD, CAD and the healthy controls (Table 2). SCL-90-R dimensions were also compared between the three groups (Table 2). There was a significant difference in anxiety between the groups (P < 0.01). The *post hoc* analysis performed with Bonferroni correction to determine from which groups this difference resulted revealed a non-significant difference between the VHD group and the CAD group, a significant difference between the VHD group and healthy controls (P < 0.01), and also between the CAD group and healthy controls (P < 0.01).

**Table 2:** Comparison of TCI and SCL-90-R Dimensions Between Patients With Valvular Heart Disease, Patients With Coronary Artery Disease, and Healthy Individuals

	VHD (N = 50)	CAD (N = 50)	Healthy controls (N = 50)	Analysis	
				F	P
TCI					
Novelty seeking	9.1 ± 3	8.3 ± 2.7	7.8 ± 2.9	2.543	0.082
Harm avoidance	9.9 ± 3.4	7.5 ± 3.6	7.3 ± 3.9	7.962	0.001
Reward dependence	9.2 ± 1.8	9.1 ± 1.7	7.9 ± 2.8	5.041	0.008
Persistence	3.4 ± 1.3	3.6 ± 1.2	3.2 ± 1.7	1.099	0.336
Self-directedness	10.5 ± 2.3	10.8 ± 2	10.2 ± 1.9	1.128	0.327
Cooperativeness	14.3 ± 3.2	15.2 ± 2.2	14.2 ± 3.4	1.924	0.15
Self-transcendence	17.6 ± 5.7	16.9 ± 5.1	15.4 ± 6	2.05	0.132
SCL-90-R					
Somatization	13.9 ± 7.2	13.5 ± 7.4	7.6 ± 5.5	13.38	< 0.001
Anxiety	8.3 ± 5.2	7.6 ± 5	4.5 ± 3	12.48	< 0.001
Obsession	9.8 ± 6.3	9 ± 5.3	6.2 ± 4	6.549	0.002
Phobia	3.2 ± 2.7	2.2 ± 2.8	1.7 ± 1.7	4.72	0.01
Interpersonal sensitivity	7.7 ± 4.1	6.7 ± 4.5	4.9 ± 3.5	5.98	0.003
Depression	14.6 ± 7.9	13.6 ± 7	7.3 ± 5.5	16.7	< 0.001
Anger	4.4 ± 3	3.8 ± 2.6	2.3 ± 1.7	9.6	< 0.001
Paranoia	6.6 ± 4.1	4.7 ± 2.9	3.8 ± 2.9	9.3	< 0.001
Positive symptom total	82.3 ± 38.5	72.5 ± 34.7	43.2 ± 24.1	18.9	< 0.001
Global severity index	3.5 ± 0.3	2.3 ± 0.2	1.1 ± 0.4	6.84	< 0.001

Obsession was detected to be significantly higher in both patient groups relative to the control group (P < 0.01). The *post hoc* analysis performed with Bonferroni correction to determine from which groups this difference resulted revealed a non-significant difference between the VHD group and the CAD group, a significant difference between the VHD group and healthy controls (P < 0.01), and also between the CAD group and healthy controls (P < 0.01).

The rate of phobia showed a significant difference among the groups (P < 0.01). It was significantly higher in the VHD group relative to the CAD group and the healthy individuals. The *post hoc* analysis performed with Bonferroni correction to determine from which groups this difference resulted revealed a significant difference between the VHD group and the CAD group (P < 0.01), and also between the VHD group and healthy controls (P < 0.01), and a statistically non-significant difference was seen between the CAD group and healthy controls (P = NS), although the rate was higher in CAD patients (P = NS).

The inter-individual sensitivity dimension showed a significant difference between the groups (P < 0.01). The inter-individual sensitivity was significantly higher in both patient groups relative to the healthy controls. The *post hoc* analysis performed with Bonferroni correction to determine from which groups this difference resulted revealed a non-significant difference between the VHD group and the CAD group, a significant difference between the VHD group and healthy controls (P < 0.01), and also between the CAD group and healthy controls (P < 0.01).

Depression showed a significant difference between the groups (P < 0.01). Depression was significantly higher in both patient groups relative to healthy individuals. The *post hoc* analysis performed with Bonferroni correction to determine from which groups this difference resulted revealed a non-significant difference between the VHD group and the CAD group.

Anger showed a significant difference between the VHD group and the other two groups (P < 0.01). The *post hoc* analysis performed with Bonferroni correction to determine from which groups this difference resulted revealed a significant difference between the VHD group and the CAD group (P = 0.01), a significant difference between the CAD group and healthy controls (P < 0.01), and also between the VHD group and healthy controls (P < 0.01).

Paranoia showed a significant difference between the groups (P < 0.01). The *post hoc* analysis performed with Bonferroni correction to determine from which groups this difference resulted revealed a significant difference between the VHD group and the CAD group (P = 0.01), a significant difference between the CAD group and healthy controls (P < 0.01), and also between the VHD group and healthy controls (P < 0.01).

### *Post hoc* analysis

For harm avoidance: VHD > CAD = HC, P = 0.004; for reward dependence: VHD = CAD > HC, P = 0.015; for somatization: VHD = CAD > HC, P < 0.001; for anxiety: VHD = CAD > HC, P < 0.001; for obsession: VHD = CAD > HC, P = 0.002; for phobia: VHD > CAD = HC, P = 0.009; for sensitization: VHD = CAD > HC, P = 0.003; for depression: VHD = CAD > HC, P < 0.001; for anger: VHD > CAD > HC, P = 0.010 and 0.001; for paranoia: VHD > CAD > HC, P = 0.015 and 0.001; positive symptom total: VHD = CAD > HC, P < 0.001; global severity index: VHD > CAD > HC, P < 0.001.

The positive total symptom level was higher both in the VHD and the CAD groups relative to the control group (P < 0.01 and P < 0.01), and similar between the VHD and CAD groups (P = 0.092).

Global severity index showed a significant difference between the groups (P < 0.01). The *post hoc* analysis performed with Bonferroni correction to determine from which groups this difference resulted revealed a significant difference between the VHD group and the CAD group (P = 0.01), a significant difference between the CAD group and healthy controls (P < 0.01), and also between the VHD group and healthy controls (P < 0.01).

### The correlation of EF and temperament-character dimensions

The correlation of EF and TCI dimensions was investigated in VHD and CAD groups and the healthy controls (Table 3). There was an inverse and weak correlation between EF and TCI-scaled persistence dimension of the temperament (r = -0.276, P = 0.052). A similar inverse correlation was found between EF and reward dependence in patients with CAD (r = -0.195, P = 0.044). In healthy controls, no correlation was observed between EF and TCI-scaled temperament dimensions (P = NS). In patients with VHD, EF demonstrated an inversely weak (r = -0.289, P = 0.042), moderate (r = -0.360, P = 0.010) and strong (r = -0.649, P < 0.001) correlation with the SCL-90-R scaled symptoms of sensitization, phobia and paranoia, respectively (Table 4). As for the patients with CAD, there was an inverse and weak correlation between EF and the SCL-90-R scaled symptoms of depression and anger (r = -0.302, P = 0.033 and r = -0.240, P = 0.054). No correlation was found between EF and the TCI-scaled temperament dimensions and SCL-90-R scaled symptom dimensions (P = NS).

**Table 3:** The Correlation of EF and TCI Dimensions in Patients With Valvular Heart Disease, Patients With Coronary Artery Disease, and Healthy Controls

	Valvular heart disease, EF (r, P)	Coronary artery disease, EF (r, P)	Healthy controls, EF (r, P)
Novelty seeking	0.019, 0.878	0.147, 0.355	0.104, 0.438
Harm avoidance	0.102, 0.455	-0.020, 0.752	0.123, 0.374
Reward dependence	-0.139, 0.314	-0.195, 0.044	-0.088, 0.507
Persistence	-0.276, 0.052	0.098, 0.422	-0.093, 0.491
Self-directedness	0.195, 0.282	0.165, 0.267	0.179, 0.186
Cooperativeness	0.162, 0.305	0.151, 0.321	0.167, 0.335
Self-transcendence	0.021, 0.813	0.042, 0.781	0.151, 0.213

r = Pearson’s correlation coefficient.

**Table 4:** The Correlation of EF and SCL-90-R Dimensions in Patients With Valvular Heart Disease, Patients With Coronary Artery Disease, and Healthy Controls

	Valvular heart disease, EF (r, P)	Coronary artery disease, EF (r, P)	Healthy controls, EF (r, P)
Somatization	-0.255, 0.071	0.006, 0.914	0.104, 0.438
Anxiety	-0.248, 0.075	0.144, 0.238	0.223, 0.094
Obsession	0.098, 0.426	0.079, 0.605	-0.121, 0.257
Phobia	-0.360, 0.010	0.120, 0.382	-0.039, 0.731
Interpersonal sensitivity	-0.289, 0.042	0.125, 0.368	0.123, 0.286
Depression	0.112, 0.202	-0.302, 0.033	0.107, 0.435
Anger	-0.183, 0.178	-0.240, 0.054	-0.187, 0.158
Paranoia	-0.649, < 0.001	-0.199, 0.213	-0.164, 0.334

In patients with VHD, EF showed an inversely weak (r = -0.289, P = 0.042), moderate (r = -0.360, P = 0.010) and strong (r = -0.649, P < 0.001) correlation with the SCL-90-R scaled symptoms of sensitization, phobia and paranoia, respectively. As for the patients with CAD, there was an inverse and weak correlation between EF and the SCL-90-R scaled symptoms of depression and anger (r = -0.302, P = 0.033 and r = -0.240, P = 0.054). No correlation was found between EF and SCL-90-R scaled symptom dimensions (P = NS) in healthy controls.

In patients with VHD, EF demonstrated an inversely weak (r = -0.289, P = 0.042), moderate (r = -0.360, P = 0.010) and strong (r = -0.649, P < 0.001) correlation with the SCL-90-R scaled symptoms of interpersonal sensitivity, phobia and paranoia, respectively. There was an inverse and weak correlation between EF and the SCL-90-R scaled symptoms of depression and anger in the patients with CAD (r = -0.302, P = 0.033 and r = -0.240, P = 0.054). No correlation was found between EF and SCL-90-R scaled symptom dimensions (P = NS) in healthy controls. No statistically significant correlation was observed between EF and the SCL-90-R scaled symptoms of somatization, anxiety and obsession (P = NS).

In patients with CAD, no correlation was found between EF and the SCL-90-R scaled symptoms of depression and anger (P = NS).

In patients with CAD, no correlation was observed between EF and the SCL-90-R scaled symptoms of phobia, inter-individual sensitivity and paranoia (P = NS).

## Discussion

We compared the VHD, CAD and healthy control groups by applying the SCL-90-R, TCI (Turkish) and by measuring their EFs. This study was performed to investigate whether the psychopathological symptoms, the temperament and character dimensions observed in patients with CAD or VHD differ among the patients and from those observed in healthy individuals, and to investigate the association between the severity of psychopathological symptoms and temperament traits, and the severity of CAD and VHD.

While mean age and gender showed similarity between the three groups, mean number of years of education was found to be lower in the VHD group. Although the incidence is decreased with improved administration of prophylaxis of streptococcal infection, VHD remains an important healthcare issue in developing countries, primarily affecting the young individuals. Its age of onset and the associated socioeconomic level may help explain the lower mean number of years of education for VHD.

A low socioeconomic level is commonly associated with psychological risk factors and unhealthy living habits. Men and women with a low socioeconomic level and/or chronic stress are potentially depressed and socially isolated [13, 14]. The mechanisms involved in the association between psychosocial factors and the increased CAD risk include unhealthy living habits (increased smoking, unhealthy diet and less physical exercise), increased use of healthcare service and lower compliance with recommendations of behavioral change or cardiac medication [14-17]. Financial difficulties to access healthcare service were shown to predict the unfavorable course following myocardial infarction (MI) [18]. Low socioeconomic level, lack of social support, presence of stressful familial and professional conditions contribute both to the risk for CAD development and deterioration of the clinical course of CAD [19]. Perception of social support by the individual has been reported to eliminate the unfavorable effects of depression [17] while lack of support enhances the unfavorable effects [20].

Depression and anxiety are psychological variables which influence morbidity and mortality in CAD and VHD. However, there are no studies in the literature investigating whether depression and anxiety differ between these two diseases. Various systematic reviews and meta-analyses report that clinical depression and depressive symptoms predict the development of CAD [21, 22] and worsen the prognosis [6, 23]. Epidemiologic studies show that panic attacks also increase the risk of cardiovascular events [24, 25]. Studies report that anxiety disorders and the presence of anxiety may predict CAD [26]. Generalized anxiety, phobic anxiety and panic attacks have been shown to worsen the course of present CAD. In patients with CAD, anxiety is also associated with increased cardiac mortality. Indeed, the presence of phobic anxiety has been associated with CAD and sudden cardiac death.

The negative effect of depression on long-term outcomes has been reported in patients who have undergone valvular surgery [6]. Major depressive disorder and post-traumatic stress disorder have been found to increase the in-hospital mortality risk following valvular surgery. The observational studies evaluating patients who underwent valvular surgery with cases of coronary bypass report an association between anxiety and increased mortality and cardiovascular morbidity.

In the present study, symptoms other than depression and anxiety such as somatization, obsession, phobia, inter-individual sensitivity, anger and paranoia, screened by SCL-90-R were systematically investigated and compared for the first time in patients with CAD and VHD. Somatization, obsession and sensitization scores were similar between CAD and VHD patients, and higher than healthy controls. As for phobia, anger and paranoia, the rates were higher in VHD cases relative to CAD cases, and higher in both patient groups compared to healthy controls. Based on these findings, as indicated in our first hypothesis, CAD and VHD may lead to different psychopathological profiles.

As is shown for the first time in our study, a correlation has been demonstrated between the severity of psychiatric symptoms and EF, a functional indicator of cardiac disease severity in cases of CAD and VHD. However, the anger scores were higher in VHD cases relative to the other two groups. The correlation between EF and anger was observed as a weak and inverse relationship. Probably, the association of anger with cardiac performance differentiates into introverted and extroverted anger. This may be considered as introverted anger is associated with paranoia. In CAD patients, the correlation between EF and anger is similar to and associated with the correlation between EF and depression.

While previous studies reported that type A behavior is more risky for VHD [27], subsequent studies did not support these findings. The meta-analyses of prospective studies have reported a non-significant correlation between type A personality and VHD events [11]. Recent studies have demonstrated that type D personality, described as anxious, hopeless, sad, socially incapable and fearful is associated with VHD [28, 29]. The risk for cardiovascular disease is found to be increased by 2.5-fold in individuals with D type personality [30]. In addition to type A and type D, there are also studies reporting that personality disorders of the B-set including histrionic, borderline, narcissistic and antisocial personality disorders are also associated with an increased VHD risk [31]. There are fewer studies investigating the same for VHD. There is no such study performed in our country. There are no studies in the literature, which compare these personality traits between these two disease groups.

While harm avoidance and reward dependence were among the temperament traits that differed between the groups, no such difference was shown for novelty seeking and persistence. Reward dependence scores were similar in the CAD and VHD groups, and higher relative to healthy controls. However, the correlation between reward dependence and EF was observed only in the CAD group. The reward dependence here may be considered to be rather based on competition and extroverted anger. In this respect, reward dependence is similar to A type personality traits. Harm avoidance is a temperament trait consistent with serotoninergic function, phobic anxiety and D type personality traits. In line with this, harm avoidance score was higher in the VHD group compared to the CAD group and the healthy individuals. Persistence is consistent with D type personality traits. In support of this finding, an inverse correlation was shown between the persistence dimension and EF in the VHD group. The phobia scores are similar to those in healthy individuals. Since the scores for anxiety and somatization were similar in the CAD and VHD cases, the differentiating point is the behavior of avoidance, i.e. phobia and harm avoidance. In healthy individuals, there was no correlation between EF and TCI-scaled temperament dimensions.

There was a weak, inverse correlation between persistence and EF in the VHD group. No difference was seen between the groups with respect to self-directedness, cooperativeness and self-transcendence sub-dimensions. Self-directedness, cooperativeness and self-transcendence sub-dimensions are sub-dimensions described for the character dimension of the personality in TCI. While temperament is an innate, relatively stable and genetically transmissible dimension of the personality, it follows a gradual character development and interacts with the education-related and environmental factors. Based on this, we may suggest that the traits differing in cardiac cases from healthy individuals are associated with the temperament dimension of the personality and the character traits do not represent such a difference. Among the temperament sub-dimensions, the lack of a difference for novelty seeking in cardiac cases compared to healthy individuals may indicate that the dopaminergic function had been preserved in these cases relative to the serotoninergic and noradrenergic function.

A meta-analysis has confirmed that anger is associated with increased cardiovascular event risk both in healthy individuals and CAD patients [32]. Inability to express anger is also reported to be important, and CAD patients who suppress their anger, are shown to have an increased risk for undesirable cardiac events [33]. A study investigating whether sudden temper and anger were acute triggers of MI reported that the relative risk for MI following a temper tantrum was increased by more than two-fold [34].

Accordingly, as we hypothesized, VHD and CAD patients differ from each other and from healthy individuals with respect to certain psychiatric symptoms and temperament traits. While EF is not associated with any psychiatric symptoms or temperament traits in healthy individuals, a weak correlation has been shown between EF and persistence and inter-individual sensitivity, a moderate correlation between EF and phobia, an inverse, strong correlation between EF and paranoia in the VHD group, and a weak, inverse correlation between reward dependence, anger and depression in the CAD group.

Our study is the first to associate psychiatric symptoms and temperament traits with cardiac disease severity; subsequent studies should focus on the continuity of this correlation and the changeability by treatment. Current studies report that CAD patients with clinically significant depression and anxiety can be safely and effectively treated with psychotherapy [35] or selective serotonin re-uptake inhibitors (SSRI) [36]. Although many studies did not reveal significant beneficial effects of them [35, 37], Davidson et al showed less common occurrence of depressive symptoms and cardiac events with them [38].

In conclusion, our study is the first to investigate the cases with VHD and CAD with respect to psychiatric symptoms and temperament traits and associate these characteristics with cardiac disease severity, and we believe that our results provide important data for the clinician’s approach to the patient, the request for psychiatric assessment and choice of treatment, if necessary. Future studies should focus on the continuity of this relationship and the modification by treatment. In fact, there are a limited number of studies reporting that routine risk factor screening and treatment of present psychiatric symptoms would be effective to prevent the development and unfavorable progression of cardiac events.

### Conclusions

VHD and CAD exhibit different psychopathological symptoms and temperament traits. There is a correlation between the aforementioned psychopathological symptoms and temperament traits, and EF.

### Limitations

The most important limitation of our present study is the relatively small sample size, so that the results of those groups may not be generalized. The cross-sectional design of this study is another important limitation. Therefore, clinical long-term follow-up with large numbers of participants is needed to before making firm conclusions.

## Abbreviations

VHD: valvular heart disease
CAD: coronary artery disease
EF: ejection fraction
SCL-R: symptom check list-revised
TCI: temperament and character inventory (Turkish)

## References

[1] Friedman M (1969). Pathogenesis of Coronary Artery Disease. New York, Mc Graw Hill.

[2] Rosenman RH, Brand RJ, Jenkins D, Friedman M, Straus R, Wurm M (1975). Coronary heart disease in Western Collaborative Group Study. Final follow-up experience of 8 1/2 years. JAMA.

[3] Taylor SE (1986). Health Psychology. New York, Random House Press.

[4] Perk J, De Backer G, Gohlke H, Graham I, Reiner Z, Verschuren M, Albus C (2012). European Guidelines on cardiovascular disease prevention in clinical practice (version 2012). The Fifth Joint Task Force of the European Society of Cardiology and Other Societies on Cardiovascular Disease Prevention in Clinical Practice (constituted by representatives of nine societies and by invited experts). Eur Heart J.

[5] Wulsin LR (2004). Is depression a major risk factor for coronary disease? A systematic review of the epidemiologic evidence. Harv Rev Psychiatry.

[6] Barth J, Schumacher M, Herrmann-Lingen C (2004). Depression as a risk factor for mortality in patients with coronary heart disease: a meta-analysis. Psychosom Med.

[7] Frasure-Smith N, Lesperance F, Talajic M (1993). Depression following myocardial infarction. Impact on 6-month survival. JAMA.

[8] Watkins LL, Blumenthal JA, Babyak MA, Davidson JR, McCants CB, O'Connor C, Sketch MH (2010). Phobic anxiety and increased risk of mortality in coronary heart disease. Psychosom Med.

[9] Roest AM, Martens EJ, de Jonge P, Denollet J (2010). Anxiety and risk of incident coronary heart disease: a meta-analysis. J Am Coll Cardiol.

[10] Roest AM, Martens EJ, Denollet J, de Jonge P (2010). Prognostic association of anxiety post myocardial infarction with mortality and new cardiac events: a meta-analysis. Psychosom Med.

[11] Myrtek M (2001). Meta-analyses of prospective studies on coronary heart disease, type A per-sonality, and hostility. Int J Cardiol.

[12] Gottdiener JS, Bednarz J, Devereux R, Gardin J, Klein A, Manning WJ, Morehead A (2004). American Society of Echocardiography recommendations for use of echocardiography in clinical trials. J Am Soc Echocardiogr.

[13] Chandola T, Britton A, Brunner E, Hemingway H, Malik M, Kumari M, Badrick E (2008). Work stress and coronary heart disease: what are the mechanisms?. Eur Heart J.

[14] Wamala SP, Mittleman MA, Schenck-Gustafsson K, Orth-Gomer K (1999). Potential expla-nations for the educational gradient in coronary heart disease: a population-based case-control study of Swedish women. Am J Public Health.

[15] Rozanski A, Blumenthal JA, Davidson KW, Saab PG, Kubzansky L (2005). The epidemiolo-gy, pathophysiology, and management of psychosocial risk factors in cardiac practice: the emerging field of behavioral cardiology. J Am Coll Cardiol.

[16] Albert MA, Glynn RJ, Buring J, Ridker PM (2006). Impact of traditional and novel risk fac-tors on the relationship between socioeconomic status and incident cardiovascular events. Circulation.

[17] Whooley MA, de Jonge P, Vittinghoff E, Otte C, Moos R, Carney RM, Ali S (2008). Depressive symptoms, health behaviors, and risk of cardiovascular events in patients with coronary heart disease. JAMA.

[18] Rahimi AR, Spertus JA, Reid KJ, Bernheim SM, Krumholz HM (2007). Financial barriers to health care and outcomes after acute myocardial infarction. JAMA.

[19] Frasure-Smith N, Lesperance F, Gravel G, Masson A, Juneau M, Talajic M, Bourassa MG (2000). Social support, depression, and mortality during the first year after myocardial infarc-tion. Circulation.

[20] Horsten M, Mittleman MA, Wamala SP, Schenck-Gustafsson K, Orth-Gomer K (2000). Depressive symptoms and lack of social integration in relation to prognosis of CHD in middle-aged women. The Stockholm Female Coronary Risk Study. Eur Heart J.

[21] Rugulies R (2002). Depression as a predictor for coronary heart disease. a review and meta-analysis. Am J Prev Med.

[22] Wulsin LR, Singal BM (2003). Do depressive symptoms increase the risk for the onset of coronary disease? A systematic quantitative review. Psychosom Med.

[23] Nicholson A, Kuper H, Hemingway H (2006). Depression as an aetiologic and prognostic fac-tor in coronary heart disease: a meta-analysis of 6362 events among 146 538 participants in 54 observational studies. Eur Heart J.

[24] Smoller JW, Pollack MH, Wassertheil-Smoller S, Jackson RD, Oberman A, Wong ND, Sheps D (2007). Panic attacks and risk of incident cardiovascular events among postmenopausal women in the Women's Health Initiative Observational Study. Arch Gen Psychiatry.

[25] Chen YH, Tsai SY, Lee HC, Lin HC (2009). Increased risk of acute myocardial infarction for patients with panic disorder: a nationwide population-based study. Psychosom Med.

[26] Janszky I, Ahnve S, Lundberg I, Hemmingsson T (2010). Early-onset depression, anxiety, and risk of subsequent coronary heart disease: 37-year follow-up of 49,321 young Swedish men. J Am Coll Cardiol.

[27] Williams RB, Haney TL, Lee KL, Kong YH, Blumenthal JA, Whalen RE (1980). Type A behavior, hostility, and coronary atherosclerosis. Psychosom Med.

[28] Denollet J, Vaes J, Brutsaert DL (2000). Inadequate response to treatment in coronary heart disease : adverse effects of type D personality and younger age on 5-year prognosis and quali-ty of life. Circulation.

[29] Denollet J, Pedersen SS, Ong AT, Erdman RA, Serruys PW, van Domburg RT (2006). Social inhibition modulates the effect of negative emotions on cardiac prognosis following percuta-neous coronary intervention in the drug-eluting stent era. Eur Heart J.

[30] Denollet J, Schiffer AA, Spek V (2010). A general propensity to psychological distress affects cardiovascular outcomes: evidence from research on the type D (distressed) personality pro-file. Circ Cardiovasc Qual Outcomes.

[31] Lee HB, Bienvenu OJ, Cho SJ, Ramsey CM, Bandeen-Roche K, Eaton WW, Nestadt G (2010). Personality disorders and traits as predictors of incident cardiovascular disease: findings from the 23-year follow-up of the Baltimore ECA study. Psychosomatics.

[32] Chida Y, Steptoe A (2009). The association of anger and hostility with future coronary heart disease: a meta-analytic review of prospective evidence. J Am Coll Cardiol.

[33] Denollet J, Gidron Y, Vrints CJ, Conraads VM (2010). Anger, suppressed anger, and risk of adverse events in patients with coronary artery disease. Am J Cardiol.

[34] Mittleman MA, Maclure M, Sherwood JB, Mulry RP, Tofler GH, Jacobs SC, Friedman R (1995). Triggering of acute myocardial infarction onset by episodes of anger. Determinants of Myocardial Infarction Onset Study Investigators. Circulation.

[35] Rollman BL, Belnap BH, LeMenager MS, Mazumdar S, Houck PR, Counihan PJ, Ka-poor WN (2009). Telephone-delivered collaborative care for treating post-CABG depression: a randomized controlled trial. JAMA.

[36] Glassman AH, O'Connor CM, Califf RM, Swedberg K, Schwartz P, Bigger JT, Krishnan KR (2002). Sertraline treatment of major depression in patients with acute MI or un-stable angina. JAMA.

[37] Berkman LF, Blumenthal J, Burg M, Carney RM, Catellier D, Cowan MJ, Czajkowski SM (2003). Effects of treating depression and low perceived social support on clinical events after myocardial infarction: the Enhancing Recovery in Coronary Heart Disease Patients (ENRICHD) Randomized Trial. JAMA.

[38] Davidson KW, Rieckmann N, Clemow L, Schwartz JE, Shimbo D, Medina V, Al-banese G (2010). Enhanced depression care for patients with acute coronary syndrome and per-sistent depressive symptoms: coronary psychosocial evaluation studies randomized controlled trial. Arch Intern Med.

